# Feasibility and techno-economic analysis of PV-battery priority grid tie system with diesel resilience: A case study

**DOI:** 10.1016/j.heliyon.2023.e19387

**Published:** 2023-08-24

**Authors:** Dessalegn Bitew Aeggegn, Takele Ferede Agajie, Yalew Gebru Workie, Baseem Khan, Armand Fopah-Lele

**Affiliations:** a School of Electrical and Computer Engineering, Debre Markos Institute of Technology, Debre Markos, Ethiopia; b Department of Electrical and Computer Engineering, Hawassa University, Ethiopia; c Department of Electrical and Electronic Engineering, Faculty of Engineering and Technology, University of Buea, PO.Box. 63, Buea, Cameroon

**Keywords:** Techno-economic analysis, PV priority grid tie, *P*–V and I–V characteristics curve

## Abstract

Ethiopia is close to the equator and has enormous potential as a solar energy resource that has yet to be realized. The country has some small-scale diesel-based power generation, and all universities and government agencies have installed standby generator sets for supplying power when the grid is interrupted. Ethiopia is a developing nation with a significant reliance on oil imports and insufficient rural electrification, which exacerbates the problem of poverty. The increased cost of oil, frequent hard currency expenditures for oil, and exaggerated maintenance costs for the generators are the main reasons to undertake this feasibility study. As the assessments show, the annual average interruption was more than 800 h in the past five years in the Debre Markos University distribution feeder. A preliminary study on the techno-economic feasibility of the existing diesel generator set and PV system with the same rated power of 500 kW is conducted in this work. As the break-even point of the economic comparison shows, the existing diesel generator is not economically feasible as compared to the proposed PV-battery priority grid tie system due to high running and service costs. The study also shows the technical feasibility of solar energy, as the *P*–V and I–V characteristic curves illustrated on a single standard solar module indicate. As the economic comparison break-even point shows, the diesel-based generator set is not economical after 5 years due to the high operational and maintenance costs. As the results show, the proposed PV/Battery Priority system with a diesel generator for resilience is more environmentally friendly, reducing the carbon footprint by 94%.

## Highlights

• Preliminary study is made on the techno-economic feasibility of the existing diesel generator set and PV system of the same rated power of 500 kW.
• PV-Battery Priority Grid Tie with diesel resilience techno-economic feasibility analysis of the selected study area is investigated.
• The diesel based standby generators compared to grid tie PV system during hazards and worst cases.

## Graphical abstract

**Figure:**
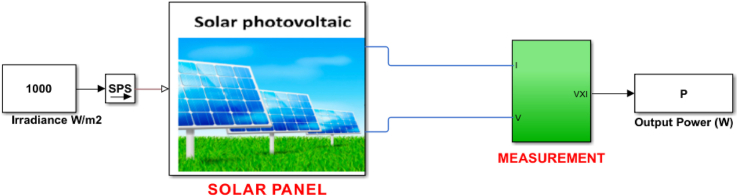

MATLAB/Simulink model of 360Wp solar module.

## 1 Introduction

The most plentiful energy source on the planet is solar energy. The amount of solar energy that strikes the surface of the Earth in a single hour is about equivalent to the energy used by all human activities in a single year [1]. The sun is a very dependable, clean, limitless, and renewable source of energy. Given the rising cost of energy, pollution of the environment, and depletion of resources, the concept of sustainable energy requires serious consideration [2,3]. In recent times, green energy (regeneration on energy) has gained much importance because of the energy resource as fossil fuel price is fluctuating. Solar PV systems are becoming more commercialized and integrated into electric power, allowing them to play an important role in the global energy mix [4,5].

Ethiopia is a developing country with high oil import demand, and more than 70% of communities lack electricity, exacerbating the poverty problem [1]. Renewable energy sources (RES), particularly distributed generation (DG), can be a supplement and even replace traditional generation. Many scholars have investigated battery- and PV-based systems as well as their connections to the national utility grid. The hybrid microgrids (HMGs) have been adapted and scaled up [3,6,7]. It has been observed that the PV and battery cloud can meet the demand without using the grid, as shown in Ref. [6]. Renewable energies such as PV systems will play a significant role in the future renewable energy technology portfolio in order to reduce carbonization in power generation. Because of increased manufacturing capacity, increased commercialization, and other efficiency improvements, battery costs continue to fall in line with PV module costs. Because of the high demand for electric vehicle manufacturing and stationary storage, battery costs are decreasing [4,[8], [9], [10]]. This is a fantastic opportunity for developing countries like Ethiopia, which has an abundance of solar energy resources.

Many types of adaptable technologies, including demand-side management, grid extension, and energy storage, can be used to control the fluctuation of solar PV energy on an hourly, daily, and annual time scale (as well as with a regional distribution) [11,12]. Several storage technologies based on electrical, chemical, thermal, and mechanical energy storage are rapidly becoming commercialized. Gandhi et al. [11,13] discussed briefly how solar energy/photovoltaic (PV) systems have become a popular energy source. Grid-tied PV systems have flourished in many countries, reaching unprecedented levels of penetration. The effects of PV on the power system and the success of the solutions are heavily influenced by PV penetration and location, as well as power system features.

The economics of grid-tie solar PV and BESS with diesel generator have been extensively researched. Numerous studies have been carried out to assess and improve the economics of PV systems without storage in relation to building type, utility rate structure, ownership alternatives, PV capacity, and PV costs [14,15]. Furthermore, efforts have been made to optimize the size and cost of behind-the-meter BESS for demand reduction as a function of tariffs [16], battery capacity [17], and load profile [18]. Recent research has focused on combining PV and BESS behind the meter [19] in grid–tie mode of operation. A thorough analysis of the mechanisms of deterioration and a modeling research on a dual-mode reversible solid oxide cell were presented in Ref. [20] by the authors. For wind-hydrogen integrated energy systems, authors in Ref. [21] reported an enhanced adaptive unscented Kalman filter (AUKF)-based power scheduling optimization technique. For the construction of Fe_2_O_3_ nanospheres with a high active interface structure for lithium-sulfur batteries, authors in Ref. [22] described an effective sulfur host based on Sn doping. The influence of consumer desire on prefabricated building developers' choices was discussed in Ref. [23]. A critical analysis of situation awareness in smart distribution networks for high-quality operation and maintenance was presented in Ref. [24] by the authors. The authors of [25] reviewed the difficulties, approaches, and uses of distributed photovoltaic data virtual collection. The authors in Ref. [26] illustrated the optimum planning approach for DG based on the steady-state security zone of the distribution network. The reliable distributed generating capacity calculation approach based on an equal power supply reliability criterion is illustrated by the authors in Ref. [27]. In seaport microgrids with cold ironing, using adaptive virtual impedance, the authors in Ref. [28] offered distributed power sharing control. For predicting the lifespan of Zn-ion batteries, researchers published an asymmetric encoder-decoder model for integrating renewable energy sources [29]. In Ref. [30], the authors presented a method for predicting the battery life of encoder-decoder fusion devices based on Gaussian process regression and improvement.

In grid tie mode of operation, the system gives priority for solar PV-BESS with a diesel generator for resilience case for all the connected loads [31]. In Refs. [32,33], studies shows the utility of on-site renewable energy but assume that all distributed energy resources are 100% reliable. Improvements in resilience due to lower fuel consumption, resulting in longer islanding durations, have also been explored. Increasing fuel stocks on-site is simple and inexpensive in most campus-like environments, and it has no significant impact on the grid-tie system performance. In all of these scenarios, neither the complete variability of a solar PV system's production nor the prospective improvement in distribution system reliability is included. As all researchers addressed the solar PV-BESS grid-tie system with a diesel generator for resilience, which is still a challenge. When there is no power from solar PV, BESS, or the national grid, the diesel generator delivers only the critical connected loads, according to this paper.

The primary goal of this paper is to assess the site's techno-economic feasibility by taking into account current diesel standby gen set expenditures, solar energy resource availability, current consumption profile, existing grid annual interruption hours, and demand side management strategies. As a result, a technical and economic comparison is made between grid-tied PV/Battery priority (with diesel resilience system) and diesel gen set standby alone.

## 2 Problem statement

In Ethiopia, most organizations and urban communities get their electricity from the national grid and/or diesel generators when the national grid fails. Electric service has significant economic and social consequences for both the utility supplying electric energy and the end users of electric service. However, power outages are becoming a daily occurrence in Debre Markos' distribution network. According to the interruption data collected over the last five years, the annual interruption of Debre Markos distribution is more than 800 h, implying that the average daily interruption is more than 2 h, requiring a backup for this period. Previously [34,35], claimed that the annual average interruption duration for each customer during a year is approximately 597.476 h. Due to these factors, Debre Markos University has many diesel generators installed and will install more in order to create a good working environment and provide quick response delivery to customers. Even if there are times when power outages occur several times per day, it is extremely difficult to tolerate power outages because they cause significant revenue loss due to the use of standby diesel generator sets and services. The diesel generator cost includes fuel logistics, fuel, and generator maintenance, and it is rising at an alarming rate. Furthermore, the diesel generator sets are placed at random, and no one knows what load they carry, resulting in resource waste due to a lack of planning and proper operation. Diesel generators with low demand have very low efficiency, especially larger generator sets, which must be loaded at least above 50% of the time, but in the case of Debre Markos University, there are 12 generators with a total capacity of 3302 kV A in the main campus that are not placed optimally, among other issues. The university has an average of 826,060 ETB for fuel only the 8 generators per year and pays unto 750,000 ETB per month for energy. Furthermore, the global problems of rapidly rising CO_2_ concentrations in the atmosphere, the greenhouse effect, and the associated severe changes in environment surface temperature and climate must be addressed and resolved quickly; and, because the institution is educational, noise from the DGS is one of the issues. After systematically identifying the aforementioned problems, one possible solution is to integrate on-grid solar PV-Battery priority distributed generation (DG) system to the DMU distribution network, because according to World Bank report 2018, Ethiopia is the second most comfortable country for renewable electric generation from Sub-Saharan countries, and Debre Markos has average solar radiation of 6.67 kW h/m^2^/day. To select on-grid solar electrification, Debre Markos University requires a careful technical and economic feasibility study for each load center [36,37]. With this information, the University's responsible higher officials and management bodies can decide on the most technically and economically viable approach for electrifying the selected connected loads and completing the project in phases.

## 3 Methodology

### 3.1 Methods*/*techniques

The PV design will be based on the critical loads and solar resources available at the site. The feasibility analysis (both technical and economic feasibility) has been investigated, and prior sample engineering economic analysis is performed based on the cost-benefit comparison of diesel generator and equivalent PV plat using real interruption data obtained from EEP Debre Markos substation over the last five years. For the purpose of comparison, the Perkins diesel generator sample data sheet is presented in Table 1, while the PV system data is presented in Table 2.

**Table 1:** Perkins diesel generator sample data sheet.

No.	Parameter	Unit	Price
1	Peak Power	kW	500
2	Fuel	Diesel	–
3	Calorific value	kWh/kg	12.7
4	Density	kg/l	0.95
5	Fuel Consumption	kWh/kg	0.25
6	Genset Price/Initial Investment	USD	181,000
7	Fuel Price	USD/l	0.6
8	Service Cost (percent)	investment/year	3%
9	Running Time (percent)	Year	10%
10	Service cost	USD/year	5430
11	Fuel consumption/year	l/year	92210.53
12	Fuel cost/year	USD/year	64547.37
13	Total operation & maintenance cost	USD/year	69977.37

**Table 2:** Proposed PV system Data.

No.	Parameter	Unit	Price
1	Peak Power	kW	500
2	Surface area	_m_^2^	3500
3	Converter	kW	500
4	Battery capacity	kWh	1500
5	PV Price with Logistics	kW	200,000
6	Converter replacement cost	USD	100,000
7	Battery Replacement cost	USD	220,000
8	Service price (percent)	USD	0.1%
9	Running time (percent)	Investment/year	100%
10	Total investment cost	USD	520,000
11	Annual Service costs	USD/year	520

### 3.2 Prior sample financial feasibility study

**Fig. 1:**
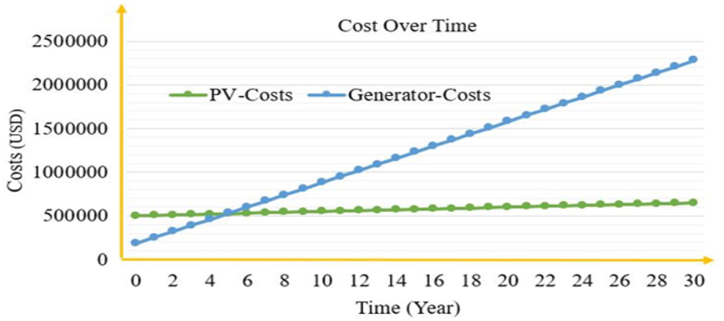
Cost comparison of Diesel generator and Solar PV.

Based on the interruption of electric power to feeder 4, which supplies the campus, the operation time of diesel generators, as well as their initial investment and operating costs, are compared with the overall solar PV plant investment for the same project life of 30 years. Fig. 1 depicts a prior sample comparison of the same rating and loading of the 500 kW diesel generator and a PV plant with all associated costs over 30 years of planning, even if the diesel generator's lifetime is not 30 years.

## 4 Results and discussion

**Fig. 2:**
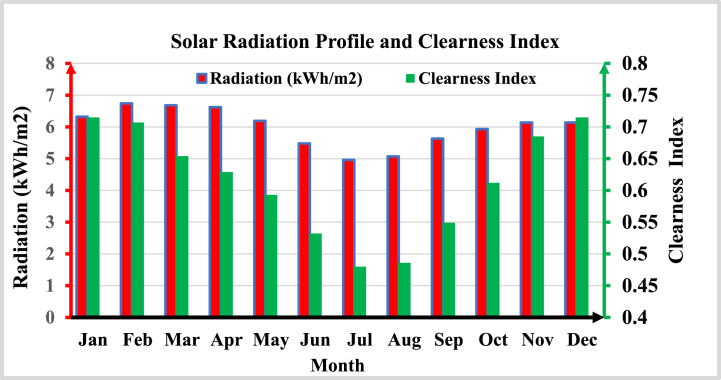
Monthly average solar irradiance profile with clearness index.

As illustrated in Fig. 1, the initial investment in the PV plant is expensive, whereas the initial cost of the diesel generator is cheap; however, the running and maintenance expenses of the diesel generator are rising at an alarming rate due to fuel costs. The PV plant has a significant initial investment but no ongoing maintenance costs. The diesel generator is no longer economically viable after 5 years; however, a PV system can be a viable project for the next 30 years, with opportunities and benefits such as income from diesel generator salvage value and annual savings from electricity billing due to the PV power plant's 70% on-grid operation. Fig. 2 presents the monthly average solar global horizontal irradiance profile with the clearness index of the site obtained from the NASA average value for over 22 years.

It is well known that both options are not economically feasible as compared to the existing grid system, but 800 h of annual interruption is a big problem to be figured out via these two alternatives and the comparison is made using a credible data collected from the owner itself and 2020 solar market from the web.

As a result of the prior sample cost comparison, the net present value of the PV system is approximately −518,232 and the NPV of the Genset system is approximately −827,527, indicating that both are not feasible projects as shown in the Appendix, but the smaller negative NPV is the better option. As a result, the PV system project may be viable as an investment to alleviate the current problem as well as the future with many upcoming opportunities. Based on the university's current revenue loss and extra expenditure for Genset service and related costs, annual savings from covered energy bills and fuel price savings can be estimated if the Genset is replaced by solar PV. The annual savings are derived from the solar PV generation of a 400 kW continuous loading plant, which covers the operating costs and energy bill. Furthermore, the plant can meet the university's off-peak and mid-peak demands, generating revenue through the utility company's feed in tariff (FiT) agreement. As a result, the project's payback period is calculated by using Equation (1) as follows [38,39]:

(1) $$\begin{array}{c} Simple\;Payback\;Period=\frac{Intial\;Investment}{Annual\;Operating\;Savings} \end{array}$$

$$Payback\;Period=\frac{USD500,000}{USD78,977/year}$$

PaybackPeriod=6.33years

Where, Annual savings is equal to annual operation and maintenance cost plus annual energy saving as shown below on Table 3.

**Table 3:** Annual costs and Savings.

Year	Fuel Consumption (l)	Price (USD)
2019	51,303	28501.67
2020	37,452	20806.67
	**Energy Bill of the University**	
2018	Annual Average consumption (kWh)	1903.53
2019	Annual Average consumption (kWh)	7022.70
2020	Annual Average consumption (kWh)	22,885.70
	Annual Saved money from energy bill	59,200
	Annual PV Service cost	520
	Annual Saved money from Genset Operation and maintenance cost	69,977
	Total annual operating Savings	128,657

Because the project's lifespan exceeds 25 years, a payback of 6.33 years is very cost-effective, even if time value of money and unforeseen issues are not considered. As a result, the cost benefit analysis clearly shows that the solar PV-Battery priority system outperforms the Genset.

The proposed PV-Battery priority grid tie system with diesel Gen set resilience is not only cost-effective, but it is also nearly emission-free, with emissions reduced by 95%.

**Fig. 3:**
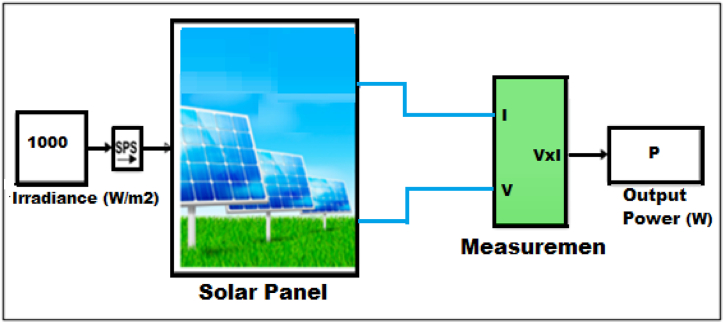
MATLAB/Simulink model of 360Wp solar module.

**Fig. 4:**
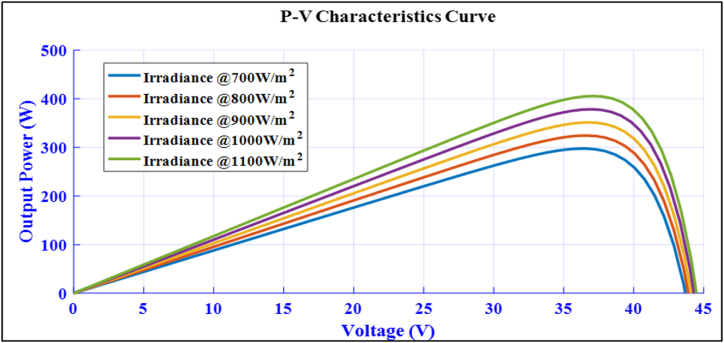
*P*–V curve of the site at different Irradiation level of a standard module.

Fig. 3 depicts the MATLAB/Simulink model of a standard module used to evaluate the module's characteristics based on the site parameters. The standard irradiance used is 1000 W/m^2^ by considering variable irradiance because of its intermittency. The model is simulated for measuring module voltage, current, power, and characteristics curves. The *P*–V curve at different irradiation levels, from 700 W/m^2^ to 1100 W/m^2^, is shown in Fig. 4 to demonstrate the module's characteristics at an STC of 25 °C.

**Fig. 5:**
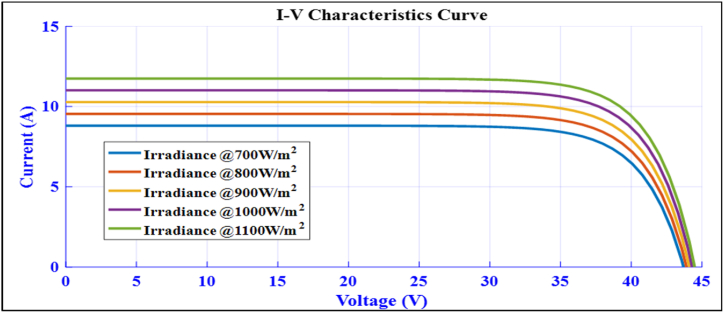
I–V curve of the site at different Irradiation level of a standard PV module.

In addition, Fig. 5 shows the I–V curves at various irradiation levels ranging from 700 W/m^2^ to 1100 W/m^2^, illustrating the module's characteristic at STC of 25 °C.

At these irradiation levels, the maximum power point consists of 72 cells and ranges from 300Wp to 400Wp [40,41]. Specification of a sample PV module:

$$P_{n}=360W,V_{mp}Vmp=33.5V,I_{mp}Imp=9.3A,V_{oc}Voc=43.2V,I_{sc}Isc=9.85A$$

This study employed a systematic investigation and collection of all relevant and credible data, which are analyzed systematically to demonstrate the cost effectiveness of the project by constructing smart PV plants in the long run to impact the country's high-tech on-grid solar energy generation.

**Fig. 6:**
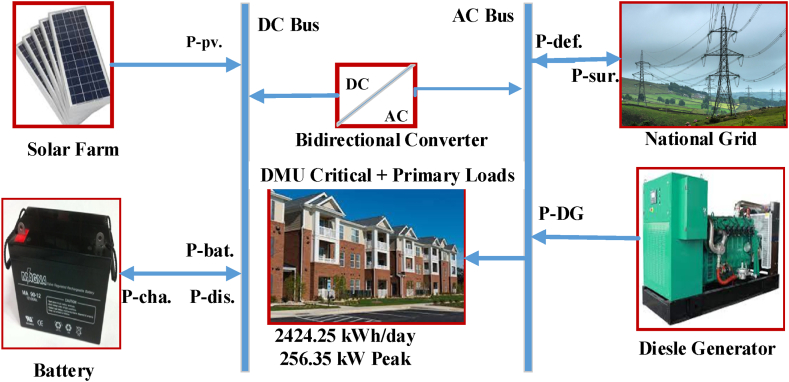
System block diagram.

Following data collection and analysis using the recommended approach, software simulations were performed using HOMER Grid, PVSYST, and MATLAB/Simulink platforms. While working on this study, some other required electrical software environments were used. Fig. 6 depicts the proposed system block diagram, which is included all of the components that must be integrated in the smart system.

Table 4 shows the daily load demand of the university based on time in use of all appliances extracted from the load survey in 24-h format. Actually, the connected loads exceed 2 MW, but the company is a commercial type, and the load factor is less than 30% based on the time in use.

**Table 4:** Daily Load Demand and Time in use.

Hour	Load (kW)	Hour	Load (kW)
0	200	12	220
1	260	13	220
2	240	14	200
3	220	15	220
4	360	16	220
5	300	17	320
6	300	18	360
7	300	19	160
8	300	20	180
9	220	21	180
10	220	22	180
11	200	23	150

**Fig. 7:**
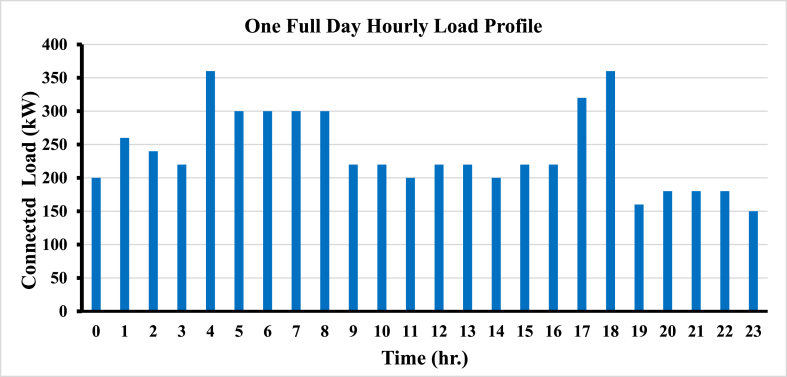
Daily load profile of the university.

Based on the amount of time that each appliance was used, Fig. 7 displays the university's daily load demand profile.

Table 5 depicts the proposed system's annually energy production and energy mix, with the PV plant accounting for the majority of the output. Moreover, during cloudy seasons, the system may receive significant energy from the utility grid. In addition, the system's energy production and consumption, demonstrating that it is fully self-sufficient and can sell energy through a Feed-in Tariff (FiT) agreement with the utility company. Earning money from a PV-Battery system during off-peak and mid-peak hours through a FiT agreement with low operating costs.

**Table 5:** Annual Energy Production and consumption.

Production	kWh/year	%	Consumption	kWh/year	%
Generic PV Array	1,389,114	96.80	Primary Load	884,852	67.7
CAT-500kW Genset	1375	0.00958	Grid Sales	442,391	32.3
Grid Purchases	44,447	3.10	Total	1,307,243	100
Total	1,434,936	100			

Table 6 summarizes the proposed project's investment cost, which includes the diesel generator set and all accessories. When compared to the utility incremental cost, the levelized energy cost is $0.05626/kWh.

**Table 6:** Economic analysis summary.

No.	Parameter	Cost (USD)
1	Total NPC	1,161,882.00
2	Levelized COE	0.005626.00
3	Operating Cost	17,502.82

Table 7 shows the annual emission of the existing standby generator set which operates for 632 h per year which releases 625,181 kg of carbon dioxide per year.

**Table 7:** Annual emission of the existing system.

No.	Parameter	Value	Units
1	Hours of Operation	632	hours/year
2	Carbon Dioxide	625,181	kg/year
3	Carbon Monoxide	0	kg/year
4	Unburned Hydrocarbons	0	kg/year
5	Particulate Matter	0	kg/year
6	Sulfur Dioxide	2200	kg/year
7	Nitrogen Oxides, NO_x_	1076	kg/year

Finally, Table 8 show the annual emission of the proposed system with annual operation of the Genset is about 7 h per year only for resilience purpose during hazards and worst conditions.

**Table 8:** Annual emission of the proposed system.

No.	Parameter	Value	Units
1	Hours of Operation	7.00	hors/year
2	Carbon Dioxide	29,175	kg/year
3	Carbon Monoxide	0	kg/year
4	Unburned Hydrocarbons	0	kg/year
5	Particulate Matter	0	kg/year
6	Sulfur Dioxide	122	kg/year
7	Nitrogen Oxides, NO_x_	59.6	kg/year

As seen from the above emission results, annual emission reduction is about 94.4% and *CO*_2_ similarly *SO*_2_ and *NO*_*x*_ gases emission is reduced by 94.5%.

Then the carbon penalty is reduced from USD31, 259 to USD1, 456.75 per annum according to the international conventions for carbon penalty.

## 5 Conclusion

Techno-economic analysis is essential for renewable energy studies. Nowadays, it is obvious that distributed generations and microgrids are coming to reality with high technical capability and appreciable cost effectiveness due to the power reliability and power quality issues in distribution networks. Grid tie PV – Battery priority system for huge consumers is recommended according to the current trends in renewable energy pricing and techno-economic analysis. This study uses credible data of the system parameters and primary data has been collected and investigated well. As the economic comparison break-even point shows, the diesel based Genset is not economical after 5 years due to the high operational and maintenance costs.

In general, this paper highlights the economic and technical issues of current diesel-based standby generator sets in universities and investigates the problems of the existing system for comparison with grid tie PV-Battery priority alternative by considering the technology's economic and technical perspectives. As the results show, the proposed PV/Battery priority system with diesel generator resilience is more environmentally friendly by reducing the carbon footprint by 94% contributed through the diesel standby generator. And the grid-tied PV-Battery system is cost-effective, with numerous advantages for both the institution and the utility company in terms of lowering exorbitant operating costs and supporting the grid.

## CRediT authorship contribution statement

**Dessalegn Bitew Aeggegn**: Conceived and designed the experiments; Performed the experiments; Analyzed and interpreted the data; Contributed reagents, materials, analysis tools or data; Wrote the paper.

**Takele Ferede Agajie**: Conceived and designed the experiments; Performed the experiments; Analyzed and interpreted the data; Contributed reagents, analysis tools or data; Wrote the paper.

**Yalew Gebru Workie:** Conceived and designed the experiments; Performed the experiments; Analyzed and interpreted the data; Contributed reagents, materials, Wrote the paper.

**Baseem Khan:** Conceived and designed the experiments; Performed the experiments; Analyzed and interpreted the data; Contributed reagents, materials, analysis tools or data; Wrote the paper.

**Armand Fopah-Lele:** Conceived and designed the experiments; Performed the experiments; Analyzed and interpreted the data; Contributed reagents, materials, analysis tools or data.

## Funding sources

There is no funding available for this research in any form.

## Declaration of competing interest

The authors declare that they have no known competing financial interests or personal relationships that could have appeared to influence the work reported in this paper.

## References

[1] E. A. Hailu, A. O. Salau, and A. J. Godebo, " Assessment of Solar Energy Potential of East Gojjam Zone Ethiopia Using Angestrom-Prescott Model,” International Journal of Engineering Research in Africa,vol. 53, pp. 171-179. DOI: 10.4028/www.scientific.net/JERA.53.171.

[2] Aderemi B.A., Chowdhury S., Olwal T.O., Abu-Mahfouz A.M. (2018). Technoeconomic feasibility of hybrid solar photovoltaic and battery energy storage power system for a mobile cellular base station in Soshanguve, South Africa. Energies.

[3] Das B.K., Alotaibi M.A., Das P., Islam M., Das S.K., Hossain M.A. (2021). Feasibility and techno-economic analysis of stand-alone and grid-connected PV/Wind/Diesel/Batt hybrid energy system: a case study. Energy Strategy Rev..

[4] Usman M., Khan M.T., Rana A.S., Ali S. (2018). Techno-economic analysis of hybrid solar-diesel-grid connected power generation system. Journal of Electrical Systems and Information Technology.

[5] Qiu Y., Yuan C., Tang J., Tang X. (2019).

[6] Adeyeye A., Tsado J., Olatomiwa L. (2018).

[7] Azerefegn T.M., Bhandari R., Ramayya A.V. (2020).

[8] Paniyil P., Powar V., Singh R., Hennigan B., Lule P., Allison M., Pumputis D. (2020). Photovoltaics-and battery-based power network as sustainable source of electric power. Energies.

[9] Imam A.A., Al-Turki Y.A. (2019). Techno-economic feasibility assessment of gridconnected PV systems for residential buildings in Saudi Arabia—a case study. Sustainability.

[10] Mubaarak S., Zhang D., Liu J., Chen Y., Wang L., Zaki S.A., Yuan R., Wu J., Zhang Y., Li M. (2020). Potential techno-economic feasibility of hybrid energy systems for electrifying various consumers in Yemen. Sustainability.

[11] Gandhi O., Kumar D.S., Rodríguez-Gallegos C.D., Srinivasan D. (2020). Review of power system impacts at high PV penetration Part I: factors limiting PV penetration. Sol. Energy.

[12] Akinyele D. (2017). Techno-economic design and performance analysis of nanogrid systems for households in energy-poor villages. Sustain. Cities Soc..

[13] Zimmer T., Rudi A., Glöser-Chahoud S., Schultmann F. (2022). Techno-economic analysis of intermediate pyrolysis with solar drying: a Chilean case study. Energies.

[14] Zhang J., Knizley A., Cho H. (2017). Investigation of existing financial incentive policies for solar photovoltaic systems in US regions. Aims Energy.

[15] Davidson C., Pieter G., Paul D., Robert M. (2015).

[16] Fisher J. Michael, Jay Apt (2017). Emissions and economics of behind-the-meter electricity storage. Environ. Sci. Technol..

[17] Yan X., Zhang X., Chen H., Xu Y., Tan C. (2014). Techno-economic and social analysis of energy storage for commercial buildings. Energy Convers. Manag..

[18] Long M., Simpkins T., Cutler D., Anderson K. (2016). North American Power Symposium (NAPS).

[19] McLaren J., Laws N., Anderson K., DiOrio N., Miller H. (2019). Solar-plus-storage economics: what works where, and why?. Electr. J..

[20] Yang Chao, Guo Ran, Jing Xiuhui, Li Ping, Yuan Jinliang, Wu Yu (2022). Degradation mechanism and modeling study on reversible solid oxide cell in dual-mode — a review. Int. J. Hydrogen Energy.

[21] Wang Y., Wen X., Gu B., Gao F. (2022). Power scheduling optimization method of wind-hydrogen integrated energy system based on the improved AUKF algorithm. Mathematics.

[22] Ren Ruiyin, Lai Fuming, Lang Xiaoshi, Lan Li, Yao Chuangang, Cai Kedi (2023). Efficient sulfur host based on Sn doping to construct Fe2O3 nanospheres with high active interface structure for lithium-sulfur batteries. Appl. Surf. Sci..

[23] Han Y., Xu X., Zhao Y., Wang X., Chen Z., Liu J. (2022). Impact of consumer preference on the decision-making of prefabricated building developers. J. Civ. Eng. Manag..

[24] Ge L., Li Y., Li Y., Yan J., Sun Y. (2022). Smart distribution network situation awareness for high-quality operation and maintenance: a brief review. Energies.

[25] Ge L., Du T., Li C., Li Y., Yan J., Rafiq M.U. (2022). Virtual collection for distributed photovoltaic data: challenges, methodologies, and applications. Energies.

[26] Sun Bing, Li Yunfei, Yuan Zeng, Chen Jiahao, Shi Jidong (2022). Optimization planning method of distributed generation based on steady-state security region of distribution network. Energy Rep..

[27] Chen Jiahao, Sun Bing, Li Yunfei, Jing Ruipeng, Yuan Zeng, Li Minghao (2022). Credible capacity calculation method of distributed generation based on equal power supply reliability criterion. Renew. Energy.

[28] Zhao P. (2022). Distributed power sharing control based on adaptive virtual impedance in seaport microgrids with cold ironing. IEEE Transactions on Transportation Electrification.

[29] Lu Siyu, Yin Zhengtong, Liao Shengjun, Yang Bo, Liu Shan, Liu Mingzhe, Yin Lirong, Zheng Wenfeng (2022). An asymmetric encoder–decoder model for Zn-ion battery lifetime prediction. Energy Rep..

[30] Dang Wei, Liao Shengjun, Yang Bo, Yin Zhengtong, Liu Mingzhe, Yin Lirong, Zheng Wenfeng (2023). An encoder-decoder fusion battery life prediction method based on Gaussian process regression and improvement. J. Energy Storage.

[31] Marqusee J., Becker W., Ericson S. (2021). Resilience and economics of microgrids with PV, battery storage, and networked diesel generators. Advances in Applied Energy.

[32] Anderson K., Laws N.D., Marr S., Lisell L., Jimenez T., Case T., Cutler D. (2018). Quantifying and monetizing renewable energy resiliency. Sustainability.

[33] Cook J., Hotchkiss E., Li X., Cruce J. (2020). Planning for the storm: considering renewable energy for critical infrastructure resilience. Journal of Emergency Management.

[34] Tsai C.-T., Beza T.M., Wu W.-B., Kuo C.-C. (2019). Optimal configuration with capacity analysis of a hybrid renewable energy and storage system for an island application. Energies.

[35] García-Vera Y.E., Dufo-López R., Bernal-Agustín J.L. (2020). Techno-economic feasibility analysis through optimization strategies and load shifting in isolated hybrid microgrids with renewable energy for the non-interconnected zone (NIZ) of Colombia. Energies.

[36] Ani V.A. (2021). Strategies for modeling and simulation of alternative energy systems for powering health facilities using HOMER application. Global Journals of Research in Engineering, 21(J3), 61–83..

[37] Olatomiwa L., Mekhilef S., Huda A.N., Sanusi K. (2015). Techno‐economic analysis of hybrid PV–diesel–battery and PV–wind–diesel–battery power systems for mobile BTS: the way forward for rural development. Energy Sci. Eng..

[38] Harmen M., Julai N., Othman A., Aznan H., Kulanthaivel G. (2020). Techno-economic analysis of a stand-alone photovoltaic-diesel hybrid system for rural area in sarawak. International Journal of Integrated Engineering.

[39] Odoi-Yorke F., Woenagnon A. (2021). Techno-economic assessment of solar PV/fuel cell hybrid power system for telecom base stations in Ghana. Cogent Engineering.

[40] Chedid R., Sawwas A. (2021). A Techno-economic feasibility study of a green energy initiative for a university campus. International Journal of Smart Grid and Clean Energy.

[41] Achirgbenda V.T., Kuhe A., Okoli K. (2020).

